# SpecTalk: Conforming IoT Implementations to Sensor Specifications

**DOI:** 10.3390/s21165260

**Published:** 2021-08-04

**Authors:** Yi-Bing Lin, Sheng-Lin Chou

**Affiliations:** 1 College of Artificial Intelligence, National Yang Ming Chiao Tung University, Hsinchu City 30010, Taiwan; 2 College of Humanities and Sciences, China Medical University, Taichung 406, Taiwan; 3 School of Computing, National Cheng Kung University, Tainan 701, Taiwan; 4 Information and Communication Research Laboratories, Industrial Technology Research Institute, Hsinchu City 30010, Taiwan; slchou@itri.org.tw

**Keywords:** acceptance test, internet of things (IoT), IoT specification, smart building, sensor, Taiwan Association of Information and Communication Standards (TAICS)

## Abstract

Due to the fast evolution of Sensor and Internet of Things (IoT) technologies, several large-scale smart city applications have been commercially developed in recent years. In these developments, the contracts are often disputed in the acceptance due to the fact that the contract specification is not clear, resulting in a great deal of discussion of the gray area. Such disputes often occur in the acceptance processes of smart buildings, mainly because most intelligent building systems are expensive and the operations of the sub-systems are very complex. This paper proposes SpecTalk, a platform that automatically generates the code to conform IoT applications to the Taiwan Association of Information and Communication Standards (TAICS) specifications. SpecTalk generates a program to accommodate the application programming interface of the IoT devices under test (DUTs). Then, the devices can be tested by SpecTalk following the TAICS data formats. We describe three types of tests: self-test, mutual-test, and visual test. A self-test involves the sensors and the actuators of the same DUT. A mutual-test involves the sensors and the actuators of different DUTs. A visual-test uses a monitoring camera to investigate the actuators of multiple DUTs. We conducted these types of tests in commercially deployed applications of smart campus constructions. Our experiments in the tests proved that SpecTalk is feasible and can effectively conform IoT implementations to TACIS specifications. We also propose a simple analytic model to select the frequency of the control signals for the input patterns in a SpecTalk test. Our study indicates that it is appropriate to select the control signal frequency, such that the inter-arrival time between two control signals is larger than 10 times the activation delay of the DUT.

## 1. Introduction

Many smart applications have been developed with the Internet of Things (IoT) technology in large-scale smart city constructions [1,2]. However, contracts of intelligent application constructions are often disputed in the acceptance due to the fact that contract specifications are not clear. The higher the level of intelligence, the greater the controversy especially when the customers and the vendors that provide the solutions have different imaginations about the functionality of smart products. Such disputes often occur during the acceptance processes of smart buildings, mainly because intelligent building systems are expensive, and the operations of the sub-systems are very complex due to the fast evolution of IoT technology.

According to Allied Market Research [3], the smart building market will reach USD 35 billion by 2020, with a compound annual growth rate of about 30 percent, driven by advances in the IoT and sensor networks [4]. A smart building typically includes different subsystems in the same building [5]. To effectively integrate and connect the subsystems, a major challenge is the deployment of the Information and Communication Technology (ICT) common trench to satisfy the needs for intelligent building system integration. These needs make it difficult for traditional contracts to standardize specific acceptance methods.

The classical method in contract law established a formula for quotation and acceptance in the 19th century, allowing buyers and sellers to reach a consensus on contracts. Formal tests of customer requirements and business processes are typically carried out at the time of acceptance, to determine whether the system meets the acceptance criteria and to enable the customer to decide whether or not to accept the system [6]. Flexible intelligent systems contracted in the traditional way are likely to be controversial for misleading conduct, and misrepresenting the right to accept. This phenomenon becomes more obvious as the intelligence elasticity of the products increases in the era of rapidly evolving ICT.

Interestingly enough, acceptance tests for complicated mobile systems have been well conducted. In mobile telecommunications systems, data formats and communication protocols are well defined by standard organizations such as the European Telecommunications Standards Institute (ETSI) and 3GPP. Following their specifications, developed systems can be systematically tested. For example, Testing and Test Control Notation (TTCN) has been used to transplant the layer-3 functions in the 4G LTE protocol stack to carry out test instruments. In [7], an implementation scheme of terminal Radio Resource Management conformance test system was implemented with TTCN-3, and the feasibility of this scheme was verified by running the designed test cases. To speed up TTCN-3 test system testing, a tool was created to visualize large test systems [8,9]. This tool was used to analyze all standardized test suites available at www.ttcn3.org, finding circular dependency and unnecessary source files in almost every test suite.

Unfortunately, there are no comparable globally accepted specifications for smart applications. In [10], Cucumber and Selenium were used to automate testing activity for a system-level test on well-known platforms such as SOASTA (Android) and Ranorex (iOS). The authors pointed out the difficulties in establishing compatibility with all smart applications due to inconsistent data formats, noting that it is challenging to ensure compatibility with many variabilities of the application platforms.

Similarly, the study in [11] investigated model-based software tests by using the Selenium library with the Graph Walker tool, which outperforms traditional software tests where JUnit and TestNG libraries were used. Comparisons of these two methods were performed and reported on parameters of the code line number, actual error detection rate, and test run-time parameters. At the smart application level, model-based software tests cannot be conducted due to inconsistent data formats. Therefore, acceptance tests of intelligent systems (smart applications) are difficult because there are no globally accepted standards to specify these systems.

At present, intelligent system-related standards, including building automation and energy-based air conditioning [12,13], have gradually involved advanced networking and IoT technologies. Since multiple systems with different communication protocols [14,15,16] and data formats [17,18] are often developed in a building, the matter of how to integrate the various systems and collect the required information is a key factor in managing building intelligence. At this stage, the market has a variety of intelligent energy management systems, independent intelligent building subsystems, and data format interfaces. It is difficult to incorporate various independent subsystems for overall monitoring and management, which affects the open and common development for future intelligent networking to heterogeneous services in smart buildings.

In light of the application situation and demand for intelligent building energy management systems, the Taiwan Association of Information and Communication Standards (TAICS) specifies the data format standard of intelligent building [13], which is applicable to the data exchange between the subsystems and the devices under the integrated system monitoring platform or the integrated operation center (IOC). However, most vendors are unable to study TAICS specifications carefully and provide Application Programming Interface (API) software that conforms to TAICS specifications. When customers request to follow TAICS standards, vendors may significantly increase the price, causing more controversy. Therefore, we design SpecTalk, an interface code generation system that makes it easy for the vendors to automatically interwork their intelligent systems to the TAICS specification-compliant platform for acceptance tests. SpecTalk does not involve data exchange within subsystems or devices. Vendors can easily integrate the APIs of their systems to the TAICS test system by using SpecTalk’s Graphical User Interface (GUI). The paper is organized as follows. Section 2 introduces TAICS; Section 3 proposes SpecTalk; Section 4 describes how SpecTalk is used to conduct three types of acceptance tests; Section 5 proposes an analytic model to select the frequency of the control signals in the input pattern of an IoT device test.

## 2. Taiwan Association of Information and Communication Standards

To highlight the state-of-the-art sensor technology in Taiwan, it is important to introduce the sensor standard efforts promoted by the Ministry of Economic Affairs (MOEA). In Taiwan, the MOEA is in charge of the 5G/IoT development. For 5G/IoT specification promotion, the mission is carried out by the Taiwan Association of Information and Communication Standards (TAICS) sponsored by the MOEA.

TAICS is an industry organization founded in June 2015 with members from industry, research, and academic organizations in Taiwan. TAICS bridges the local industry with global standard initiatives/organizations by contributing study results or consolidated consensus, and also developing the local standard or study report per request. TAICS is open to participation from all the companies/organizations with divisions in Taiwan. Seven technical committees (TCs) have been chartered, focusing on

- Advanced Mobile Communication
- Network Communication
- Device Internetworking
- Audiovisual Services and Communications
- Network and Information Security
- Intelligent Buildings ICT
- Internet of Vehicles (IoV) and Automated Driving

In Taiwan, most solution providers deliver quick and cheap IoT application constructions (such as smart buildings, smart agriculture, smart manufacturing, etc.) to the customers without conforming to any standards. To resolve this issue, this paper proposes SpecTalk, a tool that can automatically generate an interface for any IoT application to conform to the TAICS specifications. SpecTalk has been developed by Accton (one of the largest ICT companies in Taiwan—revenue of USD 1.85 billion in 2020), and aims to become the standard interworking mechanism of TAICS. SpecTalk is being used to produce receiving reports for the following smart park constructions:

- Hsinchu International AI Smart Park
- Smart Campus of China Medical University in the Shuinan Trade and Economic Park, Taichung

SpecTalk also aims to regulate agriculture-related data for the Council of Agriculture, Taiwan. We will use SpecTalk to interwork with the specifications of the following international partners:

- The Telecommunication Technology Committee (TTC), Japan
- The Telecommunications Industry Association (TIA), USA
- Institute of Electrical and Electronics Engineers (IEEE), USA
- China Communications Standards Association (CCSA), China
- European Telecommunications Standards Institute (ETSI), EU
- The Association of Radio Industries and Businesses (ARIB), Japan
- Telecommunications Standards Development Society (TSDSI), India
- Malaysian Technical Standards Forum Bhd (MTSFB), Malaysia
- Telecommunications Technology Association (TTA), Korea

## 3. The SpecTalk Architecture and Procedures

**Figure 1:**
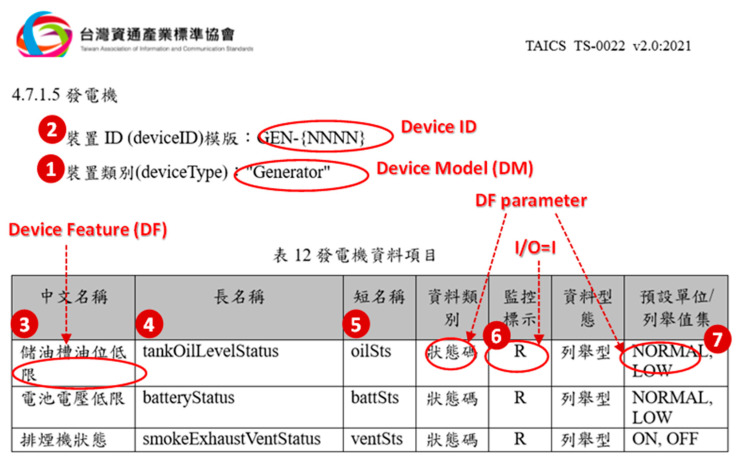
The TAICS specification for “Generator”.

All IoT-related specifications characterize an IoT device with the following fields as illustrated in Figure 1: (1) device type or device model (DM; e.g., Generator); (2) device ID (e.g., GEN-{NNNN} for a generator) and the description for the device features (DFs) including (3) DF name in Chinese; (4) DF name (e.g., tankOilLevelStatus); (5) DF abbreviation (e.g., oilSts); (6) I/O (input or output); and (7) DF parameter (e.g., status with the states {NORMAL, LOW}).

**Figure 2:**
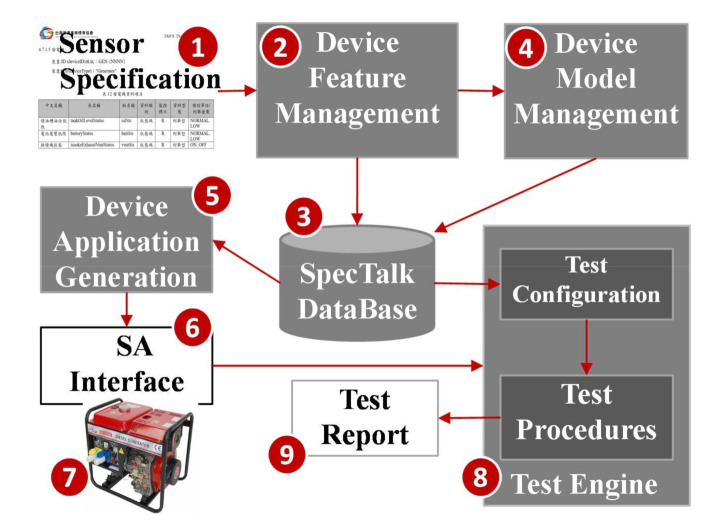
The SpecTalk approach to test an IoT device with TAICS specification.

The SpecTalk platform consists of five components: Device Feature Management (Figure 2 (2)), SpecTalk Database (Figure 2 (3)), Device Model Management (Figure 2 (4)), Device Application Generation (Figure 2 (5)), and Test Engine (Figure 2 (8)).

From the specification of an IoT device (Figure 2 (1)), we store its software configuration in the SpecTalk Database (Figure 2 (3)). The configuration is used to automatically create the Sensor and Actuator application (SA) interface (Figure 2 (6)) that binds the IoT device under test (DUT; Figure 2 (7)) to Test Engine (Figure 2 (8)). The SpecTalk Database will be maintained by a neutral third party (for example, TAICS) and can be freely accessed by both the buyers and sellers to create their contracts and acceptance test procedures for IoT applications.

### 3.1. Construction of the SpecTalk Database

**Figure 3:**
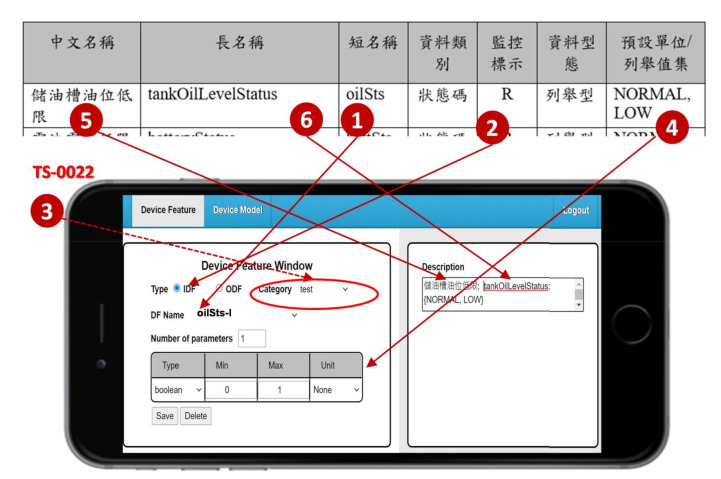
Device Feature (DF) creation.

SpecTalk is a web-based platform, which can be operated on any computing device with a browser (for example, a smartphone). In this platform, the configurations of IoT devices are stored in the SpecTalk Database through a web-based Device Feature window. We first create and store all DFs of an IoT device using the Device Feature Management (Figure 2 (2)). Consider the tank oil level status as an example. We first fill the DF name “oilSts-I” (Figure 3 (1)), then we select the DF type as “IDF” (Figure 3 (2)). Note that the DF name is appended with “-I” if it is an IDF, and is appended with “-O” if it is an output device feature (ODF). The category field is filled with the number of the specification TS-0022 (Figure 3 (3)). There is one parameter of type “Boolean” to represent the status of the oil level (Figure 3 (4)). We add extra information about the DF in the description window (Figure 3 (5),(6)). After the DF information filling is complete, the oilSts DF configuration is saved in the SpecTalk Database.

**Figure 4:**
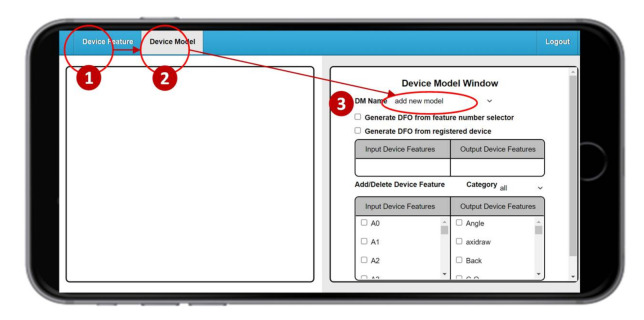
The Device Model (DM) window.

We create the DM of the IoT device using the Device Model Management by clicking “Device Model” (Figure 4 (2)). Then we jump from the Device Feature window (Figure 4 (1)) to the Device Model window (Figure 4 (3)).

**Figure 5:**
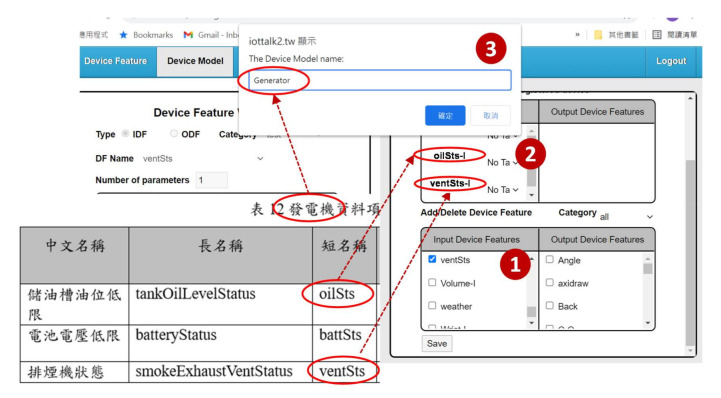
Device Feature (DF) creation.

In the Device Model window, we select the DFs from the Input/Output Device Feature lists (Figure 5 (1)). The Device Model Management automatically includes the selected DFs in the device model (Figure 5 (2)). When we click the “Save” button, a dialog box pops up for filling in the DM name (Figure 5 (3)). Then the DM “Generator” is saved in the DM list of the SpecTalk Database.

### 3.2. Generation of the SA Interface

SpecTalk automatically generates the test procedures for acceptance of a contract in two steps. At Step 1, SpecTalk generates a Sensor and Actuator (SA) interface (Figure 2 (6)). The vendor fills the API of the DUT (Figure 2 (7)) into the SA interface. At Step 2, the vendor executes the SA interface code generated by SpecTalk, and the IoT DUT is connected to the Test Engine (Figure 2 (8)). Then, SpecTalk conducts an acceptance test and automatically generates the test report (Figure 2 (9)). The Test Engine is modified from an IoT application development platform called IoTtalk [19].

**Figure 6:**
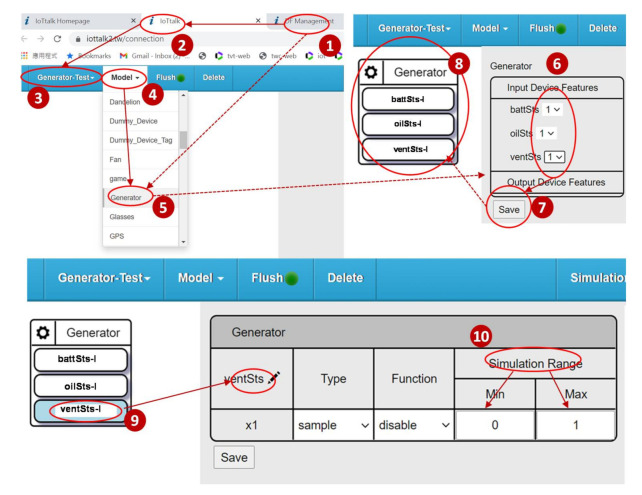
Specifying the generator under test.

By switching from the Device Feature/Model window (Figure 6 (1)) to the IoTtalk project window (Figure 6 (2)), we create the Generator–Test project (Figure 6 (3)). The Model list of the project window (Figure 6 (4)) provides the device model indexes stored in the SpecTalk Database. When we select “Generator” (Figure 6 (5)), the Generator window pops up. The features of the vendor’s generator are a subset of the Generator device model. Therefore, SpecTalk allows the selection of the DF number to set up the actual configuration of the generator under test (Figure 6 (6)). After we save the setups (Figure 6 (7)), the graphical representation of the generator under test is shown in the Project window (Figure 6 (8)). We can click the DF icon (Figure 6 (9)), and further set up the parameters of the DF, e.g., the max and the min values of the DF (Figure 6 (10)).

**Figure 7:**
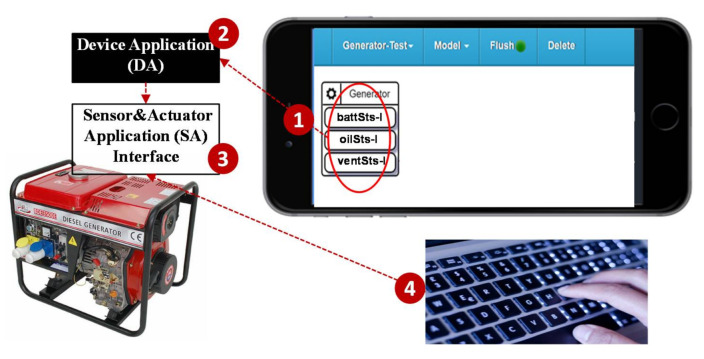
Automatic generation of the codes for the DA and the SA interface.

When we complete the IoTtalk project (Figure 7 (1)), SpecTalk automatically generates the code for Device Application (DA; Figure 7 (2)) and the incomplete code for the Sensor and Actuator Application (SA) interface (Figure 7 (3)). Then, the vendor (Figure 7 (4)) fills the API of the IoT DUT to complete the code for the SA interface.

The DA is responsible for communication between the DUT and the Test Engine. The protocols include HTTPS and MQTT. The lower-layer communications can be Ethernet, WiFi, LTE, or 5G. The code for the SA interface is listed below:

01. import time, random, requests
02. import DA
03. import Your-API
04. ServerURL = ‘https://DomainName’
05. Reg_addr = “**GEN-0012**”
06. DA.profile[‘dm_name’] = ‘**Generator**’
07. DA.profile[‘df_list’] = [‘**oilSts-I**’, ‘**battSts-I**’, ‘**ventSts-I**’,]
08. DA.profile[‘d_name’] = ‘Your-Device-Name’
09. DA.register(ServerURL, Reg_addr)
10. while True:
11. try:
12. **oilSts** _data = Your-oilSts-function
13. DA.push (‘ **oilSts-I** ‘, **oilSts** _data)
14. **battSts** _data = Your- battSts -function
15. DA.push (‘**battSts-I**’, **battSts** _data)
16. **ventSts** _data = Your- ventSts -function
17. DA.push (‘**ventSts-I**’, **ventSts** _data)
18. except Exception as e:
19. print(e)
20. if str(e).find(‘mac_addr not found:’) ! = −1:
21. print(‘Reg_addr is not found. Try to re-register.’)
22. DA.register (ServerURL, Reg_addr)
23. else:
24. print(‘Connection fails.’)
25. time.sleep(1)
26. time.sleep(0.2)

**Figure 8:**
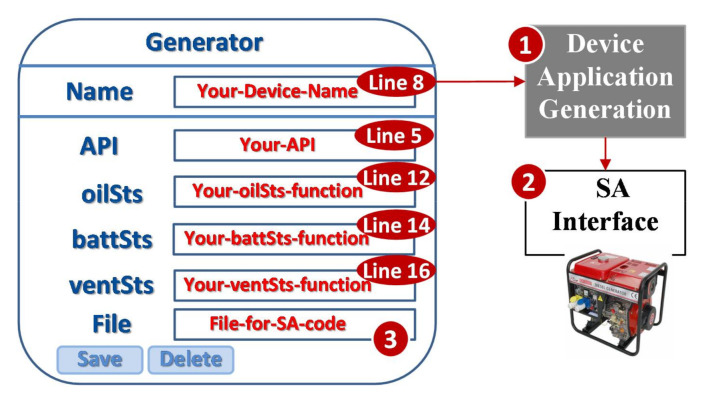
Binding the DUT and SpecTalk by creating the SA interface of “Generator”.

In the above program, the black lines with normal font are a general template for any IoT device to follow the TAICS specification. The boldface codes are generated through the steps described in Figure 6. The red parts are the codes to be typed in by the vendor (Figure 7 (4)) through the GUI for the SA interface (Figure 8). This GUI tailored for the IoT DUT is automatically generated by SpecTalk. Specifically, the values of the fields “Name”, “API”, “oilSts”, “battSts”, and “ventSts” are inserted into the code for the SA interface by the Device Application Generation (Figure 8 (1)). At Line 5, “your-API” provided by the vendor is used to access the functions for the IoT DUT (Lines 12, 14, and 16). The complete code for the SA interface (Figure 8 (2)) is saved in the file “File-for-SA-code”, specified in Figure 8 (3). Note that when a DF is created in Figure 3, the DF is used to build the device model in TAICS. Specifically, a DM and its DFs are created for the TAICS specification, and every “real” vendor device is a subset of the TAICS DM and may not have all device features specified in the TAICS DM. The actual features of the DUT are actually mapped to SpecTalk through the procedure in Figure 8. In the current implementation, Python, Java, Javascript, and C versions, the SA interface codes are available in SpecTalk.

**Figure 9:**
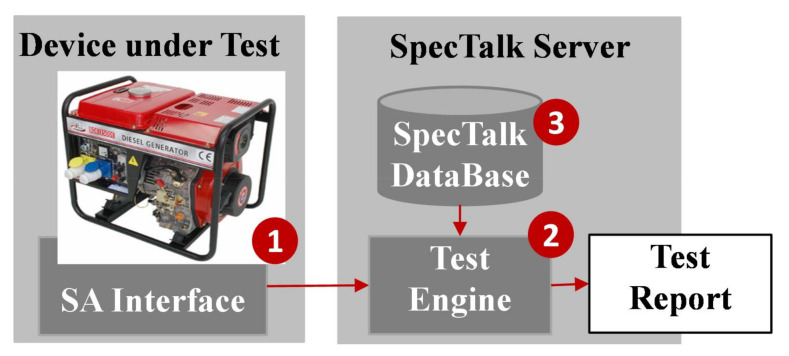
The SpecTalk test process.

At the side of the IoT DUT, the vendor executes the SA interface code (Figure 9 (1)) that registers the device to the Test Engine (Figure 9 (2)). The Test Engine retrieves the configuration of the IoT DUT from the SpecTalk Database (Figure 9 (3)) and executes the test procedures to generate the test report.

**Figure 10:**
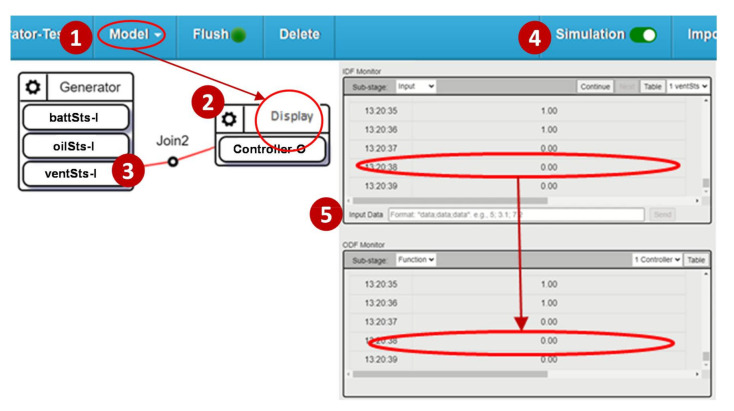
A simple SpecTalk test procedure.

A test procedure can be easily created as an IoTtalk project. A simple example is described as follows. From the Model list (Figure 10 (1)), we select “Generator” and “Display” (Figure 10 (2)). The Display prints out whatever data it receives. By dragging a line between “ventSts-I” (Figure 10 (3)) to “Controller-O”, the data will be delivered by this link (Join 2). To make sure this test procedure behaves correctly, we click the “Simulation” button (Figure 10 (4)). Following the random number generation simulation [20,21] or trace-driven emulation [22], the test results are shown in the Monitor window (Figure 10 (5)).

## 4. The SpecTalk Acceptance Tests

Three types of SpecTalk tests can be developed following the test procedure creation process similar to the one in Figure 10.

- Self-test involves the IoT DUT only
- Mutual-test involves two or more IoT DUTs
- Visual-test involves the IoT DUTs and a monitoring camera

In this section, we provide several examples to show how SpecTalk works.

### 4.1. Self-Test

**Figure 11:**
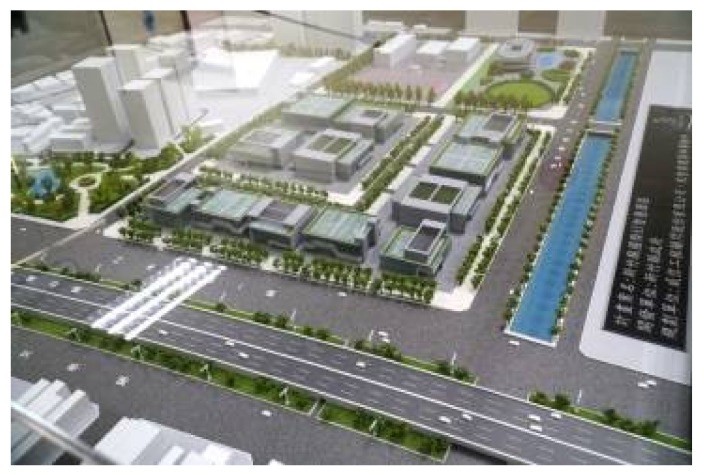
Hsinchu International AI Smart Park.

**Figure 12:**
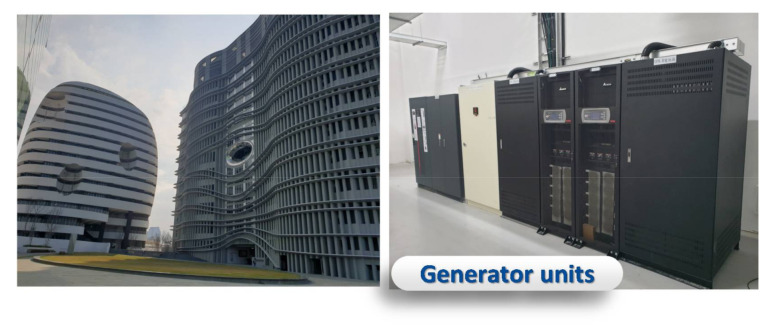
The generator units in the smart buildings of China Medical University in the Shuinan Trade and Economic Park, Taichung.

Hsinchu County is developing the Hsinchu International AI Smart Park (Figure 11), which is managed by Accton. SpecTalk will be used by Accton to verify all smart applications in the park. Most of them will be achieved through self-tests. Another example is the smart campus of China Medical University (CMU) in the Shuinan Trade and Economic Park, Taichung (Figure 12). In this subsection, we use the generator and the water-cooled chiller of the CMU smart campus as examples to show how to conduct a self-test.

**Figure 13:**
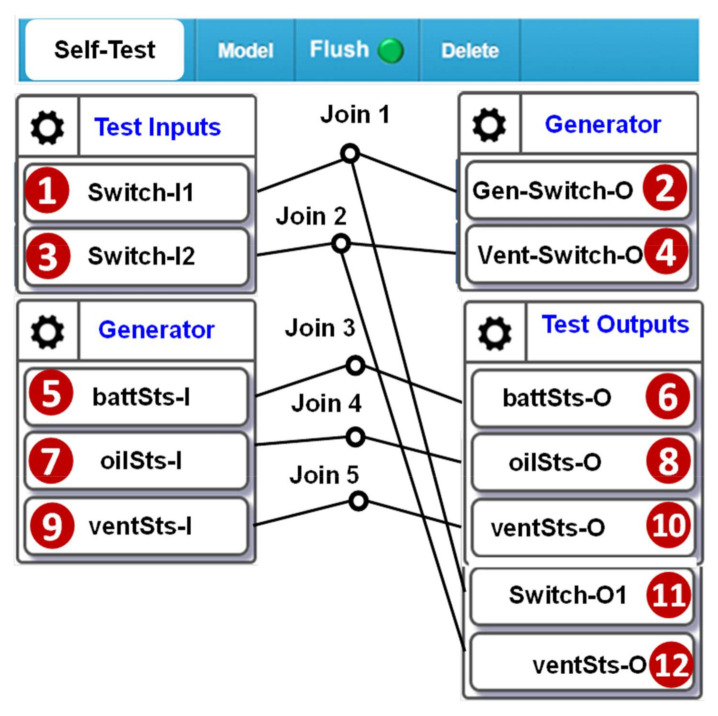
Self-test configuration for the generator.

In the IoTtalk window, we create a project “Self-Test”. Besides the three IDFs we created in Figure 6 (see Figure 13 (5),(7),(9)), we also create two output device features (ODFs): the power switch of the generator “Gen-Switch-O” (Figure 13 (2)) and the power switch of the smoke exhaust ventilator “Vent-Switch-O” (Figure 13 (4)). In the emulation mode, SpecTalk automatically generates the control signals through IDFs Switch-I1 and Switch-I2 (Figure 1 (1),(3)) to turn Gen-Switch-O and Vent-Switch-O on and off. Switch-I1 turns on the generator to see if both the battery level and oil consumption increase. The validation is performed by Test Output through battSts-O, oilSts-O and Switch-O1 (Figure 13 (6),(8),(11)). Switch-I2 tests if ventSts-I correctly indicates the ventilator operation. The validation is performed by Test Output through ventSts-O and Switch-O2 (Figure 13 (10),(12)). In the SpecTalk emulation, “Test Inputs” automatically generates sequences of test signals through random number generators. We can also use pre-stored test vectors as inputs sent to the Test Engine. The test procedure is developed manually through the GUI (which minimizes the programing effort) the first time. Then, the procedure is saved in SpecTalk to be reused for similar test cases in the future. Suppose that the oil consumption rate is given in the vendor’s datasheet. If the tank is full, then it will be consumed below the threshold in, for example, 50 min. Then, the test procedure will check if oilSts-I gives an alert after 50 min.

**Figure 14:**
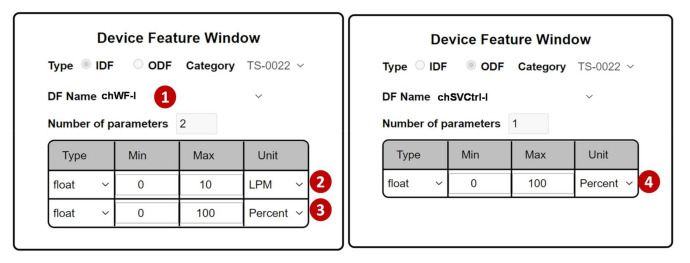
The IDF and the ODF for the water-cooled chiller.

Another example is the self-test configuration for a water-cooled chiller. In the TAICS specification, there is a two-parameter IDF “chWF-I” to indicate the water flow of the chiller. The IDF chWF-I is created through the Device Feature Management, like what we conducted in Figure 3 except that we set the parameter number to be 2 (Figure 14 (1)). The first parameter is chWQ (Figure 14 (2)), a sensor to measure the water flow rate in liters per minute (LPM). The second parameter is chSVPos (Figure 14 (3)), a sensor to measure the position of the water supply valve in percent. The ODF is chSVCtrl-O (Figure 14 (4)), the position control switch of the water supply valve. In our test, the values of chSVPos and chSVCtrl should be the same in the normal operation.

**Figure 15:**
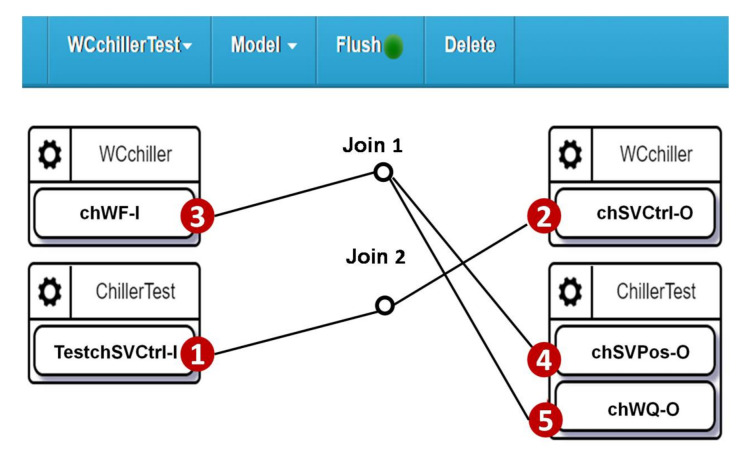
Self-test configuration for the water-cooled chiller in the smart building.

The test procedure for the water-cooled chiller is configured through the WCchillerTest project. In this project, SpecTalk automatically generates the control signals through the test input TESTchSVCtrl-I (Figure 15 (1)) to turn chSVCtrl-O (Figure 15 (2)) on and off. In Figure 15 (4),(5), we check if the position of the water level and the water flow rate measured by chWF-I (Figure 15 (3)) are consistent.

### 4.2. Mutual-Test

**Figure 16:**
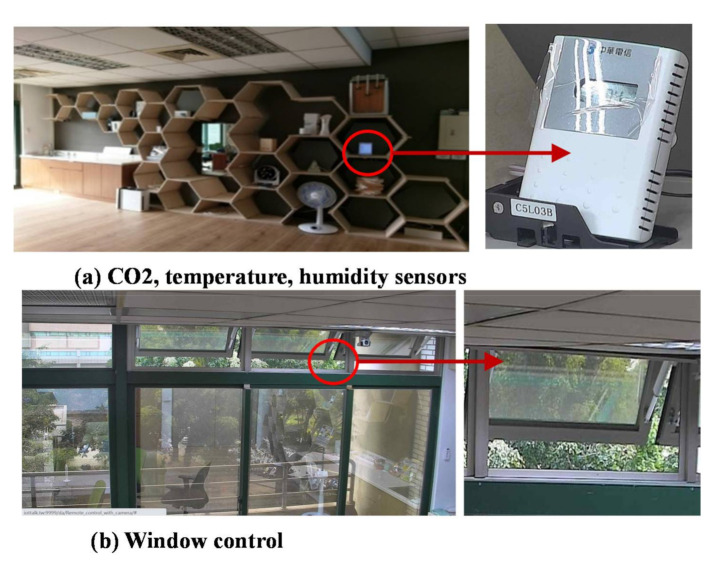
The mutual-test for window control.

A mutual-test consists of at least one sensor and one actuator under test [23]. An example is the window control where the CO_2_ sensor (Figure 16a) controls an electric window (Figure 16b) to enhance the air quality in a room [24].

**Figure 17:**
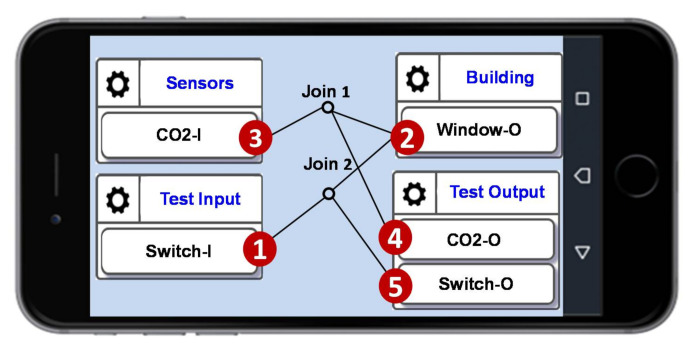
Mutual-test configuration for a CO_2_ sensor and an electric window.

The test configuration is illustrated in Figure 17. When the CO_2_ level is too high, the window is triggered to open through the control path (3)→(2). Therefore, through the test output (Figure 17 (4)) we observe if the CO_2_ level can be limited below a value. Also, in the emulation mode, SpecTalk can generate a designed sequence to turn on and off the window through Switch-I (Figure 17 (1)). Then, when Switch-O is “1”, Test Output checks if the CO_2_-O value decreases. Figure 17 also shows that a simple test procedure can be easily created through a smartphone.

**Figure 18:**
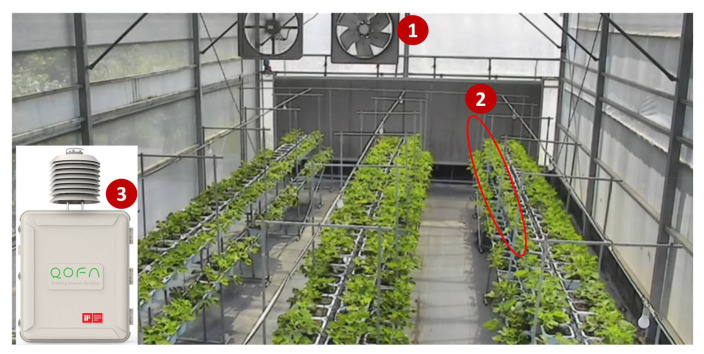
The sensors and actuators of the greenhouse on Bao Mountain, Hsinchu.

Another example is the mutual test for a greenhouse on Bao Mountain, Hsinchu. In the greenhouse, the fan and the drippers (Figure 18 (1),(2)) are controlled by the farming sensors (Figure 18 (3)).

**Figure 19:**
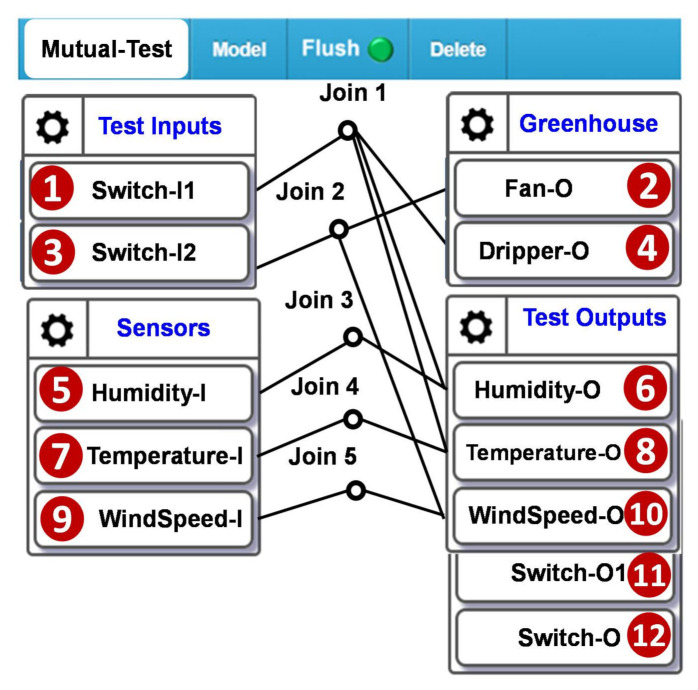
Mutual Test Configuration for the greenhouse.

We create a mutual-test configuration to investigate if the farming sensors and the actuators interact correctly. The farming sensors include those for soil humidity, soil temperature, and wind speed (Figure 19 (5),(7),(9)). The actuators include the fans and the drippers (Figure 19 (2),(4)). The test inputs (Figure 19 (1),(3)) control the actuators and serve as ground truth to the test cases.

**Figure 20:**
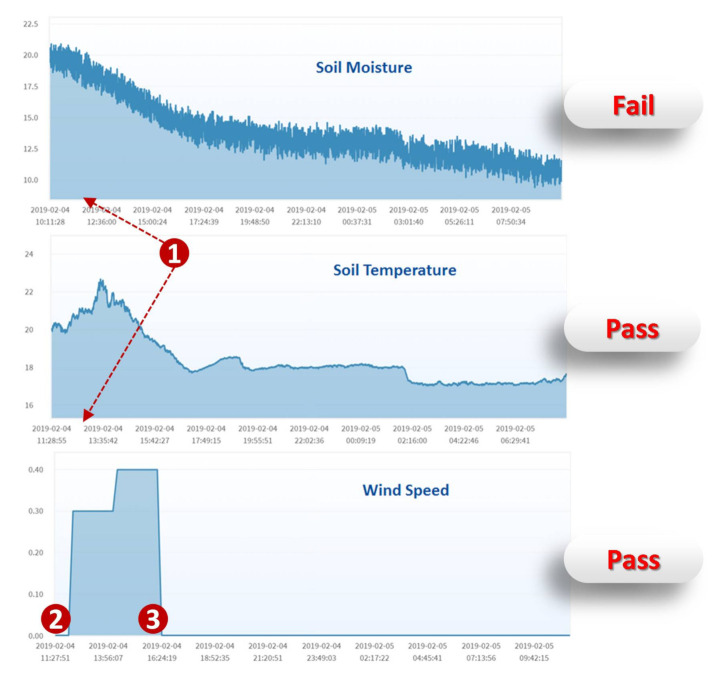
Time series charts of the greenhouse sensors.

When Switch-I1 triggered the drippers at 12 a.m. on 4 February 2019 (Figure 20 (1)), the relative humidity should increase, and the temperature should decrease. The time series charts indicated that both the humidity and the temperature decreased. Therefore, we conclude that the dripper and the temperature sensor were normal and the humidity sensor failed. Similarly, when Switch-I2 turned on the fan at 11:25 a.m. and turned it off at 2:24 p.m. (Figure 20 (2),(3)), the wind speed meter measured the correct wind speed, and therefore, both the fan and the wind speed meter were normal.

### 4.3. Visual-Test

**Figure 21:**
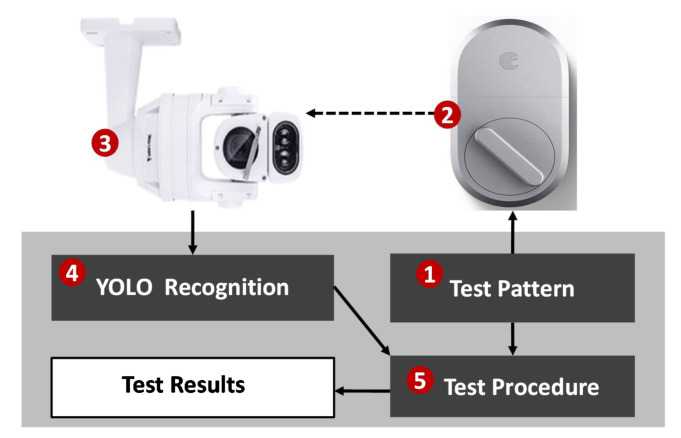
Visual test example: door lock.

When an actuator cannot be evaluated by a self-test or a mutual-test, we can observe its behavior visually. For example, when we use a smartphone to generate a sequence of test patterns (Figure 21 (1)) to lock and unlock a door, shown in Figure 21 (2), we do not know if the door is actually locked or not. Therefore, during the test period, we may use the monitoring camera (Figure 21 (3); typically installed in the ceiling) to detect the lock status. SpecTalk uses YOLO (Figure 21 (4)) for detection [25], and the results are compared with the test pattern at the test procedure (Figure 21 (5)).

**Figure 22:**
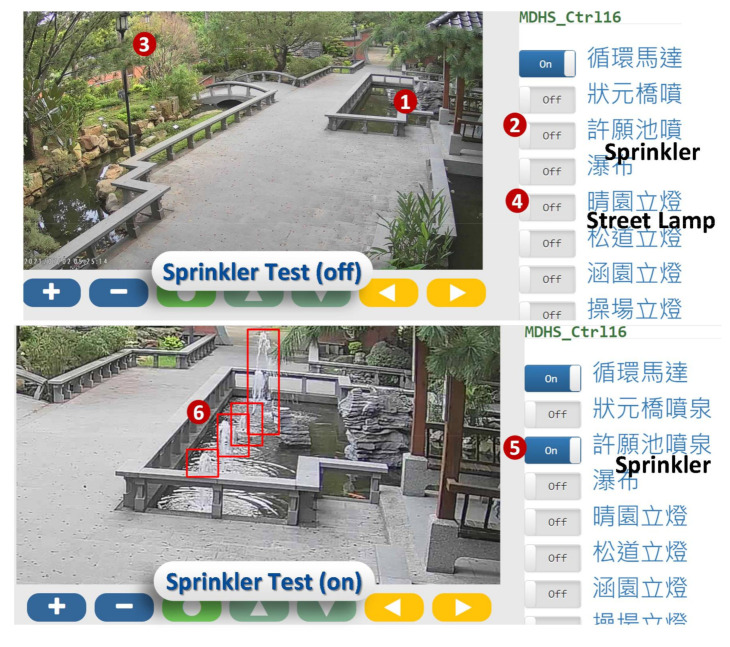
The fountain in the Ming Der High School, Taichung.

Another visual test was conducted in a fountain of the Ming Der High School, Taichung. In this example, the monitoring camera is used to test multiple actuators such as sprinklers (Figure 22 (1)) and street lamps (Figure 22 (3)). The test inputs can be automatically generated or manually triggered through the control board (Figure 22 (2),(4)). When we turn on the sprinkler control (Figure 22 (5)), the YOLO Recognition is expected to detect four water columns (Figure 22 (6)).

**Figure 23:**
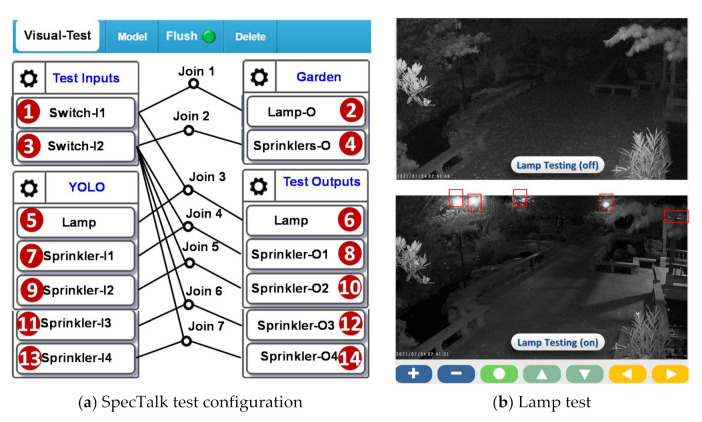
The visual-test configuration for a fountain in the Ming Der High School, Taichung.

The SpecTalk test configuration is illustrated in Figure 23a, which involves (3)→(4), (3)→(8), (3)→(10), (3)→(12), (3)→(14), (7)→(8), (9)→(10), (11)→(12), and (13)→(14). The lamp test is conducted at night (Figure 23b), where five lamps are expected to be detected by YOLO. The SpecTalk test configuration involves (1)→(2), (1)→(6) and (5)→(6). In the sprinkler test, YOLO reports the statuses of individual sprinklers. Alternatively, in the lamp test, YOLO does not report the status of a specific street lamp.

**Figure 24:**
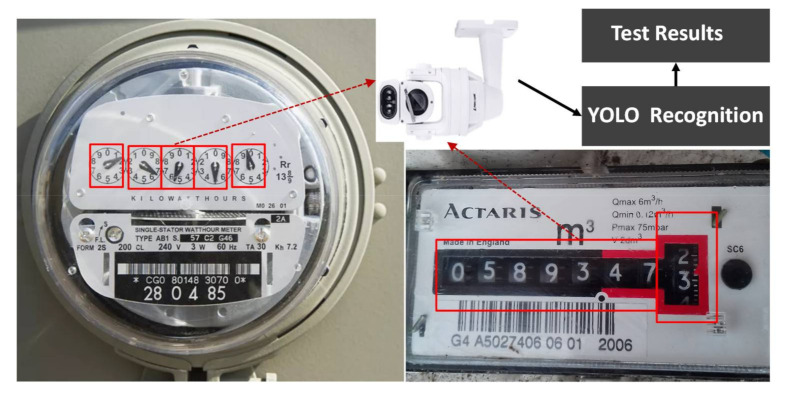
Visual-tests for analog meters.

Visual-tests are particularly useful for retrieving data from analog meters as illustrated in Figure 24. In visual-tests, YOLO already knows the specific spots under test from the monitoring camera, and therefore can accurately detect the changes on these spots. We can conduct multiple tests simultaneously in one video screen, for example, for the sprinkler test and the lamp test in the Ming Der High School (Figure 22 and Figure 23).

## 5. Selection of the Frequency of the Control Signals in the IoT Device Test

**Figure 25:**
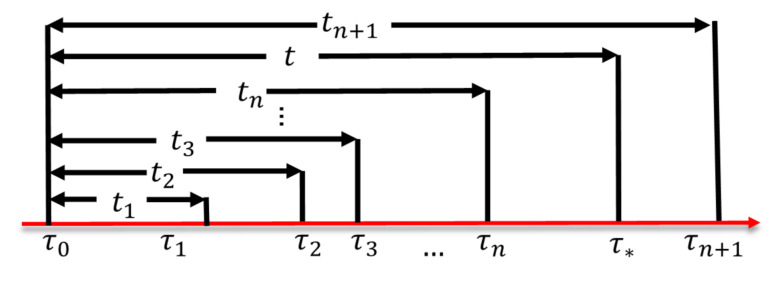
The timing diagram.

While one may attempt to quickly complete an IoT device test by selecting a high frequency of the control signal generation for the input pattern, such selection may result in incorrect test results. Consider the test in Figure 19, where the dripper actions are triggered by the control signals issued from Switch-I1. Suppose that the dripper motor receives the “on” signal at $\tau_{0}$ in Figure 25, and the motor is turned on at $\tau_{*}$, then the activation delay of the motor is $t=\tau_{*}-\tau_{0}$. The characteristics (the mean and the variance) of t for the actuator may be provided in the vendor’s data sheet. Otherwise, we need to measure the operation delays. Such measurements can be simply conducted using the Network Time Protocol. If Switch-I1 continues to send the control signals at $\tau_{1}$, $\tau_{2}$,$\;\ldots ,\;\tau_{n}$,$\;\tau_{n+1}$, where $\tau_{n}\leq \tau_{*}\leq \tau_{n+1}$, then the signals arrive at $\tau_{2}$,$\;\ldots ,\;\tau_{n}$ will be ignored and the motor will execute the instruction arrives at $\tau_{n+1}$. Therefore, the inter-arrival times of the control signals should be longer than t to avoid producing incorrect test results.

Let $t_{i}=\tau_{i}-\tau_{0}$. We typically generate Poisson input patterns. That is, the inter-arrival times of the control signals have an Erlang distribution with the expected value E[$t_{i}]=i/\lambda$. Then the density function $g\left(t_{i}\right)$ has an Erlang density function of

$$g\left(t_{i}\right)=\frac{\lambda^{i}t_{i}{}^{i-1}e^{-\lambda t_{i}}}{\left(i-1\right)!}$$

Let t have a general density function $f\left(t\right)$, then

(1) $$\begin{array}{cc} \mathrm{Pr}[t>t_{n}] & =\int_{t=0}^{\infty }f\left(t\right)\int_{t_{n}=t}^{\infty }\left[\frac{\lambda^{n}t_{n}{}^{n-1}e^{-\lambda t_{n}}}{\left(n-1\right)!}\right]dt_{n}dt\\ & =\int_{t=0}^{\infty }f\left(t\right)\overset{n-1}{\underset{i=0}{\sum }}\left[\frac{\lambda^{i}t^{i}e^{-\lambda t}}{i!}\right]dt\\ & =\overset{n-1}{\underset{i=0}{\sum }}\left(\frac{\lambda^{i}}{i!}\right)\int_{t=0}^{\infty }t^{i}f\left(t\right)e^{-\lambda t}dt \end{array}$$

Let $f^{*}\left(s\right)=\int_{t=0}^{\infty }f\left(t\right)e^{-\lambda t}\;dt$ be the Laplace Transform of $f\left(t\right)$. Then, from the frequency-domain derivative of the Laplace Transform, Equation (1) is re-written as

(2) $$\mathrm{Pr}\left[t>t_{n}\right]=\overset{n-1}{\underset{i=0}{\sum }}\left(-\frac{\lambda^{i}}{i!}\right){\left.\left[\frac{f^{*\left(i\right)}\left(s\right)}{ds^{i}}\right]\right|}_{s=\lambda }$$

From Equation (2), we have

(3) $$\mathrm{Pr}\left[t_{n}<t<t_{n+1}\right]=\left(-\frac{\lambda^{n}}{n!}\right){\left.\left[\frac{f^{*\left(n\right)}\left(s\right)}{ds^{i}}\right]\right|}_{s=\lambda }$$

Equation (3) gives the probability that *n* − 1 consecutive control signals are ignored by the IoT DUT. The probability that no control signal is lost is $\mathrm{Pr}\left[t<t_{2}\right]$, which is expressed as

(4) $$\begin{array}{c} \mathrm{Pr}\left[t>t_{2}\right]=1-\mathrm{Pr}\left[t<t_{2}\right]\\ =1-f^{*}\left(\lambda \right)+\lambda {\left.\left[\frac{f^{*}\left(s\right)}{ds}\right]\right|}_{s=\lambda } \end{array}$$

If $f\left(t\right)$ is a Gamma density function with the shape parameter α and the scale parameter β, then its Laplace transform is

(5) $$f^{*}\left(s\right)=\frac{\beta^{\alpha }}{{\left(s+\beta \right)}^{\alpha }}$$

The Gamma distribution is considered because this distribution is often used in computer and communication modeling [26,27]. Substitute Equation (5) to Equation (3) to yield

(6) $$\mathrm{Pr}\left[t_{n}<t<t_{n+1}\right]=\left(\begin{array}{c} \alpha +n\\ n \end{array}\right){\left(\frac{\lambda }{\lambda +\beta }\right)}^{n}{\left(\frac{\beta }{\lambda +\beta }\right)}^{\alpha }$$

Substitute Equation (5) to Equation (4) to yield

(7) $$\mathrm{Pr}\left[t>t_{2}\right]=1-\frac{\beta^{\alpha }}{{\left(\lambda +\beta \right)}^{\alpha }}-\frac{\alpha \lambda \beta^{\alpha }}{{\left(\lambda +\beta \right)}^{\alpha +1}}$$

In the Gamma distribution, a small α implies a large variance of t, and the operation of the DUT is not stable. We have

$$\underset{\alpha \to 0}{\lim}\frac{\beta^{\alpha }}{{\left(\lambda +\beta \right)}^{\alpha }}=1\;\mathrm{and}\;\;\underset{\alpha \to 0}{\lim}\frac{\alpha \lambda \beta^{\alpha }}{{\left(\lambda +\beta \right)}^{\alpha +1}}=0$$

Therefore, from Equation (7),

(8) $$\underset{\alpha \to 0}{\lim}\mathrm{Pr}\left[t>t_{2}\right]=0$$

Equation (8) states that if the activation time of the DUT has a high variance, then the control signals are likely to be lost during the test. For the mean value analysis [28], we assume that α=1, then E[t]=1/β and Equation (6) is re-written as

(9) $$\begin{array}{c} \mathrm{Pr}\left[t>t_{2}\right]=1-\frac{\beta }{\lambda +\beta }-\frac{\lambda \beta }{{\left(\lambda +\beta \right)}^{2}}\\ ={\left(\frac{\lambda }{\lambda +\beta }\right)}^{2}={\left(\frac{E\left[t\right]}{E\left[t_{1}\right]+E\left[t\right]}\right)}^{2} \end{array}$$

If we select E[$t_{1}]=10E\left[t\right]$, then $\mathrm{Pr}\left[t>t_{2}\right]>0.99$, and almost no control signals will be lost. Note that in Equation (6), if α→0,

$$\underset{\alpha \to 0}{\lim}\mathrm{Pr}\left[t_{n}<t<t_{n+1}\right]={\left(\frac{\lambda }{\lambda +\beta }\right)}^{n}$$

which is a general form of Equation (8). When α→∞, Equation (6) states that $\underset{\alpha \to \infty }{\lim}\mathrm{Pr}\left[t_{n}<t<t_{n+1}\right]=0$, and $\underset{\alpha \to \infty }{\lim}\mathrm{Pr}\left[t>t_{2}\right]=1$. The above analysis indicates that it is appropriate to select the control signal frequency such that the inter-arrival time between two control signals is larger than 10 times of the activation delay of the DUT. Note that the residual probability of the wrong IoT device communication sequence still exists. Such probability is affected by the variance of operation (switching) time of the controlled actuator.

## 6. Conclusions

This paper proposed SpecTalk, a platform that automatically generates the code to conform IoT applications to TAICS specifications. Specifically, SpecTalk generates a program to accommodate the API of the IoT DUTs. Then, the device can be tested by SpecTalk following the TAICS data formats. We described three types of tests: self-test, mutual-test, and visual test. A self-test involves the sensors and the actuators of the same DUT. A mutual-test involves the sensors and the actuators of different DUTs. A visual-test uses a monitoring camera to investigate the actuators of multiple DUTs. We conducted these types of tests in the Hsinchu International AI Smart Park, and the greenhouse on Bao Mountain, Hsinchu. We also exercised acceptance tests of the smart buildings in the China Medical University in Shuinan Trade and Economic Park, and the fountain of the Ming Der High School, Taichung. Our experiments in the tests proved that SpecTalk is feasible and can effectively conform IoT application implementation to TACIS specifications. We also proposed a simple analytic model to select the frequency of control signals for the input patterns in a SpecTalk test. Our study indicates that it is appropriate to select the control signal frequency such that the inter-arrival time between two control signals is larger than 10 times the activation delay of the DUT. In this paper, we conducted a primary derivation of the probability aiming to produce a simple close form. Right now, we do not have enough practical examples to investigate a secondary detailed derivation (which may result in tedious non-close form equations). The secondary derivation will be considered in future work.

Based on SpecTalk, we can interwork TACIS with all smart applications conforming to the specifications developed by TTC (Japan), TIA (USA), IEEE (USA), CCSA (China), ETSI (EU), ARIB (Japan), TSDSI (India), MTSFB (Malaysia), and TTA (Korea). For example, we can use the procedure in Figure 8 to connect all devices that conform to the ETSI TS 103 410-4 specification. Through this simple process, TACIS data formats can be transformed to the data formats specified by other standard organizations.
